# Evaluating student’s ability to assess treatment claims: validating a German version of the Claim Evaluation Tools

**DOI:** 10.1186/s12889-022-14700-w

**Published:** 2023-02-07

**Authors:** Jana Hinneburg, Barbara Gasteiger-Klicpera, Jürgen Kasper, Julia Lühnen, Katharina Maitz, Thomas Martens, Anke Steckelberg

**Affiliations:** 1 Institute for Health and Nursing Science, Medical Faculty, Martin Luther University Halle-Wittenberg, Halle (Saale), Germany; 2 Inclusive Education Unit, Institute of Education Research and Teacher Education, University of Graz, Graz, Austria; 3 Department of Nursing and Health Promotion, Faculty of Health Sciences, OsloMet Metropolitan University, Oslo, Norway; 4 Faculty of Human Sciences, MSH Medical School Hamburg University of Applied Sciences and Medical University, Hamburg, Germany; 5 Magdeburger Str. 8, 06112 Halle (Saale), Germany

**Keywords:** Evidence-based medicine, Health education, Health literacy, Critical thinking, Treatment claims, Informed choices, Rasch analysis, Validation, Probabilistic test theory

## Abstract

**Background:**

The Claim Evaluation Tools measure the ability to assess claims about treatment effects. The aim of this study was to adapt the German item sets to the target group of secondary school students (aged 11 to 16 years, grade 6 to 10) and to validate them accordingly. The scale’s reliability and validity using Rasch’s probabilistic test theory should be determined.

**Methods:**

We conducted a sequential mixed-method study comprising three stages: contextualisation and adaption of the items (stage 1), piloting of the item sets using qualitative interviews (stage 2) and a construct validation by testing the unidimensional Rasch scalability for each item set after data collection in one secondary school in Germany and two secondary schools in Austria. We explored summary and individual fit statistics and performed a distractor analysis (stage 3).

**Results:**

Secondary school students (n = 6) and their teachers (n = 5) participated in qualitative interviews in Germany. The qualitative interviews identified the need for minor modifications (e.g. reducing thematic repetitions, changing the order of the items). The data of 598 German and Austrian secondary school students were included to test for Rasch scalability. Rasch analyses showed acceptable overall model fit. Distractor analyses suggested that model fit could be improved by simplifying the text in the scenarios, removing and editing response options of some items.

**Conclusion:**

After the revision of some items, the questionnaires are suitable to evaluate secondary school students’ ability to assess health claims. A future goal is to increase the pool of items being translated and tested.

## Background

Nowadays, people are confronted with a flood of information, which is sometimes conflicting, misleading and questionable – especially in the current SARS-CoV-2 pandemic. Claims about treatment effects can be found in the media, advertisements, health information and through communication with physicians, friends and family. Claims about the benefits and harms of treatments (or a health-related action) describe what outcomes are caused by the treatment [1], e.g. “Not smoking can make you happy!” [2]. Despite the flood of health information available, most information does not fulfil the quality criteria for evidence-based health information [3–5]. Citizens need to be able to assess the reliability of these claims by thinking critically and making decisions whether to believe them or not [6, 7]. Therefore, people need good health literacy in order to be prepared to think critically, assess the evidence, critically appraise information and consequently to be able to make good choices. Otherwise, they might suffer unnecessarily and waste resources by getting therapies (or other interventions) that are sometimes harmful or ineffective, and might thus miss helpful, effective treatments [1].

To meet this challenge, the international, multidisciplinary working group of the Informed Health Choices (IHC) project aims to enable people to recognise reliable claims about treatment effects and make informed health choices by developing, evaluating and disseminating resources [8, 9]. To date, the IHC Network includes over 120 people from over 30 countries [9].

The ability to assess treatment claims is one aspect of critical health literacy [10]. A comprehensive definition of health literacy is as follows: *“Health literacy is linked to literacy and entails people’s knowledge, motivation and competences to access, understand, appraise, and apply health information in order to make judgments and take decisions in everyday life concerning healthcare, disease prevention and health promotion to maintain or improve quality of life during the life course.”* [11]. According to this definition, the competent management of health-related information is a major aspect of health literacy [12]. Nutbeam (2000) distinguished a three-level hierarchy of health literacy (functional, interactive and critical health literacy), where critical health literacy is the highest level [13]. Critical health literacy implies that citizens are enabled to critically appraise the quality of identified health information [10] and to engage in informed decision-making. Unfortunately, the search for high-quality health information is challenging, e.g. because quality criteria are not considered in the ranking algorithms of the internet search engines [14]. Furthermore, familiar and commercial online information sources are often rated as being trustworthy [15].

The promotion of (critical) health literacy should start at the latest in primary school [16–19]. Therefore, the members of the IHC project developed 49 plain language Key Concepts based on the concept of evidence-based medicine [20] (originally 32 [21]), which can be embedded in education for citizens of all ages and which are thought to evaluate the trustworthiness of claims. The Key Concepts, first published in 2015, are considered as evolving and were therefore revised over the years according to feedback, suggestions and a systematic review [7, 22]. In 2022, the most current version was published [20]. The Key Concepts can help people to beware of untrustworthy treatment claims, check the evidence from treatment comparisons and make well-informed health choices [17]. This means, for example, that personal experiences or anecdotes are an unreliable basis for most claims (e.g. “the drug helped me”). Moreover, fair comparisons are necessary to estimate a treatment effect. Furthermore, it is important to weigh up the expected benefits and harms of an intervention.

The Key Concepts are not a learning resource itself but can inform the development of learning resources, curricula and evaluation tools [7, 17, 23]. The IHC working group developed, pilot tested and evaluated teaching materials for primary as well as secondary schools in different languages [1, 9, 17]. Moreover, a database for learning and teaching resources on evidence-based health care was provided, which includes the IHC learning resources [24, 25]. Further pilot testing and evaluation studies are underway [9]. Currently, the Key Concepts are not taught in a structured manner in German or Austrian secondary schools, because they are not integrated into the curricula.

Furthermore, the Claim Evaluation Tools were developed iteratively based on the Key Concepts using qualitative and quantitative methods, providing an objective generic instrument to measure the ability to assess claims about treatment effects [26] (as one important aspect of critical health literacy). They are freely available for non-commercial use via the Testing Treatments interactive website [27]. This is a pool of multiple-choice questions which are assigned to the Key Concepts and can be used as outcome measures in trials [17], to evaluate trainings and for competence measurement in cross-sectional studies [28].

The first validation study using Rasch analysis applied to the Claim Evaluation Tools was published in 2017 [17]. In recent years, the items have been translated, contextualised, pilot tested and validated in different countries like Uganda, Norway, China, Mexico, Croatia and Germany [17, 26, 28–32]. In addition, the Claim Evaluation Tools have been used in trials to assess outcomes. In Uganda, 24 multiple-choice questions from the Claim Evaluation Tools item bank were used as an outcome measure in a cluster-randomised trial evaluating an intervention designed to teach primary school children to assess claims about treatment effects [33].

In 2016, the German working group translated 68 items addressing 22 of the original Key Concepts [21, 26] from the Claim Evaluation Tools item bank and conducted a validation study. The data collection was carried out online as well as paper-based at schools and universities in Germany. The sample of 805 people included students from vocational grammar schools, trainees in health care occupations, nursing students, students in health sciences and citizens between 16 and 52 years (mean 22.4). The study showed that some of the items were too easy to solve so that item difficulty needed to be increased by adjusting task difficulty or distractors. Furthermore, distractor analysis revealed that some distractors could be recognised as incorrect too easily [32]. Two items were removed because they showed an underfit.

The aim of the current study was to adapt the German item sets to the target group of secondary school students and to validate them accordingly. The scale’s reliability and validity in terms of Rasch’s probabilistic test theory should be determined. Rasch’s theory and approach to measure a trait is based on the assumption of an underlying dimension representing both item difficulties and individual capacity [34]. In the case that the Rasch model describes the empirical data well, the person’s capacity can be determined by the probability of solving items. Items can be randomly selected from an item bank, but items that are located close to the person’s position on the underlying dimension will lead to a better estimation of the person’s capacity [35]. The Rasch model implies the scale’s homogeneity, which means the order of the items with regard to difficulty is stable between persons and groups of persons. The investigation of the validity of the instrument in the current study was done by determining the overall fit of the model to the empirical data and also by investigating the fit of single items. Proving the instrument’s Rasch scalability provides, amongst other advantages, chances to standardise the measurement of critical health literacy across studies and groups by using items from the item bank [36].

## Methods

Using mixed methods, the study was designed as a sequence of three stages: contextualisation and adaption of the items (stage 1), piloting of the item sets using qualitative interviews with secondary school students and their teachers (stage 2) and a construct validation by testing the unidimensional Rasch scalability (stage 3). The study was conducted in Austria (November 2018 - February 2019) and in Germany (October 2018 - April 2019) and approved by the Lower Saxony State Board of Education (Niedersächsische Landesschulbehörde) in Germany and the Provincial School Board for Styria (Bildungsdirektion Steiermark) in Austria.

### Stage 1: contextualisation and adaptation

First, we contextualised the German items from the Claim Evaluation Tools item bank in autumn 2018. The items contained examples relating primarily to Sub-Saharan Africa or developing countries in general. The adaptation included changes concerning the language used and item topics to achieve a better fit for the German and Austrian target group as well as the cultural context in order to prevent potential measurement biases. The division into three sets of multiple-choice items from the previous German validation study was retained. The revised version of the item sets for secondary school students comprised 66 items addressing 22 Key Concepts [21]. One item was included in all three item sets (resulting in 68 items in total).

### Stage 2: piloting

In this substudy, we piloted the items in qualitative interviews using the think aloud method [37] with secondary school students and their teachers. We carried out interviews with students to explore potential barriers towards responding to the questions, readability, comprehension and acceptance (e.g. terminology, instructions and format). Knowledge about possible barriers could prevent potential measurement biases [9].

The aim of the interviews with teachers was to obtain an expert assessment of the items in relation to the target group of secondary school students. Barriers regarding the reading ability of the target group, possibly unknown or difficult terms and relevant examples for the target group were to be identified. In addition, we checked whether the German gender regulation (e.g. use of alternative forms for masculine and feminine gender) has a negative impact on readability.

#### Setting and sample

The interviews with secondary school students (aged 11 to 16 years, grade 6 to 10) and their teachers were carried out in a secondary school in Germany. A teacher was asked to choose diverse students regarding age, gender and performance. Students and teachers participated voluntarily in the interviews. The number of interviews was checked consistently with regard to whether data saturation was deemed achieved. In addition, the Austrian working group of the project *Health literacy and diversity for secondary school students (HeLi-D)* [38] (Box 1) and one Austrian teacher checked for needs for adjustment considering the two marginally varying languages.

Box 1: Health literacy and diversity for secondary school students (HeLi-D) project. HeLi-D is an adaptive digital training programme to promote digital health literacy in students aged 12 to 15 years. It addresses health-related information by means of stories, informational texts, quizzes and research tasks. The programme has an integrated assessment that measures the students’ reading skills and adaptively assigns content in four difficulty levels. The programme was developed in participatory workshops with students and evaluated in a controlled longitudinal study with 1,113 students. The results showed an increase in the students’ health knowledge and internet-related health literacy [38].

**Data collection and procedure**

An information sheet was provided for teachers, parents and students prior to the interviews. Data collection was carried out by JH in Germany. Before the interviews, the socio-demographic data sex, age and grade of the students and sex, age and teaching experience of the teachers were surveyed. Data were anonymised for publication. Approximately 30 min were scheduled for each student interview and 2.5 h for each teacher interview. Students were asked to read one of the three item sets and teachers all three item sets. The teacher interviews were conducted prior to the student interviews. We asked the participants to read the items and share their thoughts. In the process, the interviewer could ask questions about the participants’ thoughts.

**Data analysis**

JH made notes on proposed changes directly on the printouts of the items. JH and AS discussed and agreed on the suggested revisions. In the case of disagreement, JL was consulted. Subsequently, the items were revised accordingly. Revisions included a linguistic and content adjustment, and were carried out in an iterative process between the interviews.

### Stage 3: construct validation

To test basic assumptions made in the construction of the questionnaires and relevant for application, this substudy aimed at investigating the dimensional structure of data collected with the three revised item sets in Germany and Austria.

#### Setting and sample

Recruitment of secondary school students (aged 11 to 16 years, grade 6 to 10) was performed in Germany and Austria. Data collection involved a convenient sample and was carried out at a combined general and intermediate secondary school in Germany and at two general secondary schools in Austria. In Austria, the validation was embedded in the project HeLi-D [38]. The three item sets were assigned to secondary school students who had not received any training related to the Key Concepts in both countries.

There is no established rule for determining the sample size of a survey purposing on performing Rasch analyses. As in other statistical analyses, small samples are associated with less precise estimates, less powerful fit analysis and less robust estimates [39]. We aimed to include approximately 250 completed questionnaires per item set.

#### Data collection and procedure

Students participated voluntarily in the study. We provided an information sheet for parents and students prior to the study. Data collection was anonymous and we only surveyed age, sex and grade. It was carried out online in Austria and paper-based in Germany. Decisions about how to assign the questionnaires were made according to what was considered feasible in the local environments. In Germany, the anonymity was ensured by using a box in which questionnaires were put after completion. No written informed consent was required. In Austria, parents gave written informed consent to the students’ participation in the HeLi-D project and related research activities. All of the data collected during the project were stored and processed in anonymised form. Withdrawal from the study was possible before or during the study without giving reasons and without any disadvantage for those concerned. Data collection was carried out by JH in Germany and a team of researchers and university students in Austria. The students in each grade level were randomly assigned to one of the three item sets. In Germany, the older students from the 9th and 10th grades filled out two item sets.

#### Data analysis

Socio-demographic data were analysed descriptively using IBM SPSS Statistics version 27. Validity of the three adjusted item sets was approached by Rasch analyses using WINMIRA 2001 version 1.45 [40]. Measurement using Rasch theory or item response theory is based on the assumption of a latent dimension representing both item difficulty and persons’ capacities with regard to the given construct. The analysis determines whether and to what extent the scale properties allow it to be used for reliable assessment of the individual’s capacity level. This would also imply the scale’s ability to precisely localise a person’s level of capacity to a sufficient likelihood. The following properties were calculated to appraise the quality of the scales:

- Item difficulty: Scales designed for Rasch-based assessment use to provide much variability regarding item difficulty. It is important to consider the item difficulty with regard to its distribution over the scale and test feasibility considerations.
- Two estimators of reliability (Anova and Andrich’s): Anova reliability works according to Cronbach’s alpha in classical test theory. Andrich’s reliability is considered more important for appraisal of a scale in terms of the Rasch theory. Andrich’s approach to reliability focuses on measurement of persons and not on item statistics, i.e. on the quality of the separation of persons [41]. Therefore, Andrich’s reliability is used as the person separation index in this study. Values higher than 0.60 are considered moderate, higher than 0.80 good and higher than 0.90 excellent. In this study, values higher than 0.7 were considered acceptable.
- Q indices display whether an empirical pattern from a single item fits the parameter estimation according to the Rasch model, representing item specific indicators of model fit. Using a p-value, Q indices express empirical deviation from the estimation of single items in one of two directions: an item underfit implies that the item’s localisation cannot be interpreted properly, because the chance for solving these items deviates for some people or subgroups, e.g. the order of item difficulties can be different for this subgroup. Indication of overfit for single items is less problematic. It just means that the item characteristic curve, ideally represented as a sigma curve, is seen as a clear step from not being able to solve the items to solving it by 100%. Such an item behaves according to the Guttman model [42], but the order of item difficulties is not disturbed.
- Pearson’s coefficients of a bootstrap test: Bootstrap approaches [43, 44] use the model’s parameter estimation to generate multiple random data samples. The empirically generated sample is compared to the parameter generated samples. A significant p-value for analysis of model fit by bootstrap approach indicates that the empirical sample does not fit into the range of parameter generated samples, thus implying a poor model fit. As different parameters might contradict each other, appraisal of model fit in terms of homogenous Rasch scalability is not necessarily easy.

In case of misfit, it was planned to perform distractor analyses to inform a discourse about removing or adjusting items or distractors on the basis of distributions of frequencies calculated using SPSS.

## Results

### Stage 1: contextualisation and adaption

The contextualisation and adaptation included changes like the use of the familiar German form of “you” instead of the formal one, the application of the German gender regulation, the exchange of some unfamiliar names and the deletion of unknown terms like “Kyogero” (herbal bath used in Uganda). Examples used were also changed (e.g. milk with honey instead of water with honey, washing gel instead of soap). In addition, some sentences were simplified.

### Stage 2: piloting

#### Participants

We performed five interviews with teachers and six interviews with students. All the participants completed the interviews that lasted approximately one hour with the teachers and 20 min with the students. The mean age of the teachers was 48.2 years (range 29–65 years), all were female and had 3 to 43 years (on average 18 years) teaching experience. The mean age of the 7th and 9th grade students was 13.5 years (range 12–15 years). Four of the six students were female.

#### Results of teachers

The teachers in particular named terms (e.g. study, conventional) which they suspected the secondary school students might not be familiar with. In principle, concerns were raised regarding the length of the item sets with regard to the students’ ability to concentrate. In addition, the participants made suggestions to improve the readability, e.g. by shortening sentences. On the advice of the teachers, the order of the items was partially modified. Easily readable items were chosen to get started with, followed by text-heavier items to counteract the declining ability to concentrate. Furthermore, thematic repetitions within the item sets were avoided by adjusting the examples. The language check by the Austrian working group and one secondary school teacher revealed the need for two modifications (e.g. the German term for beetroot is not common in Austria).

#### Results of students

None of the terms classified as critical by the teachers was unknown to the students. All in all, the students rated the instrument as legible and easy to understand. The length of the instrument was also assessed as manageable. As a result of the interviews, which revealed the need for a longer reading time for the students, the required processing time was adapted from 20 to 30 [32] to 30–45 min. Neither teachers nor students criticised the German gender regulation with regard to its impact on readability.

### Stage 3: construct validation

#### Participants

598 students (Germany n = 254, Austria n = 344) completed at least one item set. 125 of the German participants completed two sets. The completion of one item set lasted a maximum of 30 min. 49% of the students were female (n = 293). The mean age of the secondary school students was 13.5 years (range 11–18 years). 150 (25%) students from the 6th, 173 (29%) from the 7th, 150 (25%) from the 8th, 63 (11%) from the 9th and 60 (10%) from the 10th grade participated. There were 3.5% or less missing or incorrect responses per item set.

#### Results from Rasch analyses

Table 1 shows an overview of fit statistics by item set, separate for each country and in total.

**Table 1:** Overview of fit statistics by country and in total

	Germany			Austria			Total		
**Item set**	1	2	3	1	2	3	1	2	3
**Items (n)**	23	23	22	23	23	22	23	23	22
**Persons (n)**	121	126	116	115	117	111	236	243	227
**Item difficulty**	0.58	0.58	0.51	0.46	0.45	0.48	0.53	0.52	0.49
**Cronbach’s alpha**	0.78	0.76	0.72	0.70	0.73	0.75	0.77	0.77	0.74
**Person separation index**^**a**^	0.72	0.69	0.62	0.57	0.64	0.66	0.70	0.71	0.64
**Items with underfit (overfit)**	1 (1)	3 (0)	0 (0)	3 (0)	3 (0)	7(1)	7 (3)	3 (3)	1 (1)
**Overall model fit: p-values for bootstrap estimation**^**b**^	0.03*	0.13	0.43	0.08	0.65	0.53	0.03*	0.18	0.73

*statistically significant ^a^Andrich’s reliability ^b^Pearson parameter

The total number of filled-in item sets was 236 for item set 1, 243 for item set 2 and 227 for item set 3

Item difficulties were moderate: 0.53 averaged over item set 1, 0.52 over item set 2 and 0.49 over item set 3. The analysis of how the three item sets fit into the model of homogenous Rasch scales revealed the following results: the person separation indices were 0.70, 0.71 and 0.64 for the item sets 1, 2 and 3 in total. These values indicate moderate reliability. Cronbach’s alpha was acceptable (> 0.7 for all item sets).

Rasch analyses revealed the need for adjustment of several items to optimise the item fit into the Rasch model. 11 out of 66 items showed an underfit (seven in item set 1, three in item set 2 and one in item set 3). Moreover, seven items indicated overfit (three in item sets 1, three in set 2 and one in item set 3). The bootstrap approach to model fit turned out to be significant (p = 0.03) for item set 1, indicating lacking goodness of fit to the Rasch model, and unobtrusive for the other two item sets, indicating a sufficient model fit. Distractor analyses and the associated discourse suggested that many items could be improved by simplifying the text in the scenarios, removing and editing response options. One item was removed because it was classified as very difficult for the target group to understand and two other items from this Key Concept were still contained in the item sets. All other items with underfit were adjusted in terms of content.

## Discussion

We contextualised, adapted and validated the German items for the target group of secondary school students. The validation study showed that most of the items of the Claim Evaluation Tools can be used for evaluating secondary school students’ ability to assess treatment claims since they have acceptable model fit. However, some items needed to be improved by simplifying the text in the scenarios, removing or revising response options.

***Strengths and limitations***.

An important strength of this study is that we used Rasch analysis for psychometric testing and optimisation which allows to examine the level of skills being measured and to identify variability in measurement precision [45]. The item sets can easily be administered and are time-saving since only one of the three item sets must be used to measure the ability to assess treatment claims. Moreover, the Claim Evaluation Tools directly connect to the Key Concepts which serve as a definition of the skills to be acquired [45]. The inclusion of the target group in the development of health literacy measures has proven to be a sound method to improve the quality of instruments [18]. Therefore, qualitative interviews were conducted to explore potential barriers, readability, comprehension and acceptance of the item sets by the students and to obtain an expert assessment in relation to the target group by the teachers.

This study also has limitations. Although a teacher was asked to choose diverse students for the interviews, we cannot rule out that better-performing students were selected and therefore overrepresented. Since the sample of the construct validation was a convenient one, it is not representative of all secondary school students in Germany and Austria, especially due to the heterogeneity caused by the federal education system in Germany with different types of secondary schools. The limited number of participating schools and the homogeneity regarding school type (general and intermediate secondary schools but no academic secondary schools included) limit the generalisability of the results. Moreover, the item sets were only tested in two German speaking countries. They were not validated in Switzerland. The inclusion of 250 questionnaires per item set was not quite achieved. Therefore, further robust studies should confirm the results. How the items function in other settings (e.g. with existing cultural differences) is unknown. We did not include gender and migration background in the analysis. Furthermore, the pool of items being translated and tested might be increased because the list of Key Concepts and corresponding items has been extended. However, some Key Concepts might be too difficult to be understood and applied by younger secondary school students. Since the item bank was updated, mirroring the last changes to the Key Concept list, the translated items need to be re-arranged so that they match the latest version [9, 20]. However, the update has no impact on the methods used or the results of this validation study.

***Comparison with other studies***.

So far, items from the Claim Evaluation Tools item bank have been translated and validated in several settings and languages [17, 26, 28–31]. The findings of our study are comparable to those of the other validation studies in terms of reliability and confirm the value of the German item sets as a flexible tool for measuring the ability of secondary school students to assess treatment claims objectively. In the validation study with a sample of people from Uganda and Norway, 17 out of 88 items (19%) were identified with a poor model fit [17]. In our study 11 out of 66 items (17%) were identified.

There are at least 202 validated health literacy measures available. They differ concerning measured domains (e.g. numeracy, comprehension), context (e.g. generic, disease-specific), approach for tool development (e.g. Rasch), administration time, validation study (e.g. sample, modes of administration), language and assessment (objective/performance-based or subjective/self-reported) [46]. Most instruments are guided by classical test theory and only a small number by modern measurement theories like the item response theory [36]. A systematic review on generic health literacy measurement instruments for children and adolescents identified fifteen instruments including seven objective (performance-based tests), seven subjective (self-reporting) and one mixed-method measure [18]. Two of these instruments measure critical health literacy – the *Critical Health Competence test (CHC test)* and the *All Aspects of Health Literacy Scale (AAHLS)*. The CHC test is also based on the concept of evidence-based medicine and uses objective measures to assess the actual performance. Likewise, Rasch analysis was used and the test was validated in a sample of secondary school students (grade 10 and 11) and university students [10]. The AAHLS is based on subjective measurement using self-report and covers functional, communicative and critical health literacy [18]. The validity of self-reporting has often been criticised, for instance because of measuring self-efficacy rather than health literacy [18]. The *European Health Literacy Survey (HLS-EU)*, which is also based on self-reporting [47], is often used to report the deficient health literacy of the German population, despite the lack of appropriateness and relevance of its items [48, 49]. Furthermore, the applicability of the HLS-EU is limited for measuring general health literacy among adolescents [50]. In general, there is a lack of evidence regarding child and adolescent health literacy and the varying understanding of health literacy hampers the comparison of different instruments and results [18].

***Implications and future research***.

The aim of the IHC project is teaching students to think critically about health claims and choices as a major aspect of (critical) health literacy. Learning materials for primary and secondary schools have already been developed and will be evaluated in randomised trials in Kenya, Rwanda and Uganda in 2022 [9]. A remaining challenge is the training of teachers who may not possess the competences required for teaching the Key Concepts. They probably need support in the form of guidance or teach the teacher courses. Critical health literacy has to be taught across subjects since it includes subjects like Math, Biology and English. Therefore, concepts and resources for cross-subject teaching like team teaching must be organised. Moreover, it would be reasonable to teach and learn the Key Concepts using a spiral curriculum reinforcing previously learned content while introducing new concepts [7]. Many schools in Germany and Austria still have knowledge-based instead of competence-based curricula, which complicates the acquisition of health literacy.

A further step is the translation of the IHC learning resources into German so that the item sets could be used as an outcome measure in a randomised trial, evaluating the effects of training on the ability of secondary school children to assess claims about treatment effects. Especially in the light of the SARS-CoV-2 pandemic, the digitalisation of the learning materials seems to be reasonable. At the moment the Claim Evaluation Tools measure only a part of critical health literacy. Additionally, the Key Concepts and consequently the Claim Evaluation Tools could be expanded covering diagnostic accuracy claims, for example. In principle, items measuring functional and interactive health literacy could be included additionally.

## Conclusion

After the revision of some items, the item sets are suitable for being used as an outcome measure to evaluate secondary school students’ ability to assess treatment claims and for objective competence measurement in cross-sectional studies. It is the only Rasch-scaled instrument available in the German-speaking countries for this age group, which is sufficiently reliable and can be used as an objective measure of critical health literacy. A future goal is to increase the pool of items being translated and tested.

## Data Availability

The datasets generated and analysed during the current study are available from the corresponding author on request. All items from the Claim Evaluation Tools item bank are available upon request for non-commercial use.

## Abbreviations

AAHLS: All Aspects of Health Literacy Scale
CHC test: Critical Health Competence test
HeLi-D: Health literacy and diversity for secondary school students
HLS-EU: European Health Literacy Survey
IHC: Informed Health Choices
